# A Comparison of Red Fluorescent Proteins to Model DNA Vaccine Expression by Whole Animal *In Vivo* Imaging

**DOI:** 10.1371/journal.pone.0130375

**Published:** 2015-06-19

**Authors:** Ekaterina Kinnear, Lisa J. Caproni, John S. Tregoning

**Affiliations:** 1 Mucosal Infection & Immunity Group, Section of Virology, Imperial College London, St Mary’s Campus, London, United Kingdom; 2 Touchlight Genetics Ltd., Leatherhead, Surrey, United Kingdom; Commissariat a l'Energie Atomique(cea), FRANCE

## Abstract

DNA vaccines can be manufactured cheaply, easily and rapidly and have performed well in pre-clinical animal studies. However, clinical trials have so far been disappointing, failing to evoke a strong immune response, possibly due to poor antigen expression. To improve antigen expression, improved technology to monitor DNA vaccine transfection efficiency is required. In the current study, we compared plasmid encoded tdTomato, mCherry, Katushka, tdKatushka2 and luciferase as reporter proteins for whole animal *in vivo* imaging. The intramuscular, subcutaneous and tattooing routes were compared and electroporation was used to enhance expression. We observed that overall, fluorescent proteins were not a good tool to assess expression from DNA plasmids, with a highly heterogeneous response between animals. Of the proteins used, intramuscular delivery of DNA encoding either tdTomato or luciferase gave the clearest signal, with some Katushka and tdKatushka2 signal observed. Subcutaneous delivery was weakly visible and nothing was observed following DNA tattooing. DNA encoding haemagglutinin was used to determine whether immune responses mirrored visible expression levels. A protective immune response against H1N1 influenza was induced by all routes, even after a single dose of DNA, though qualitative differences were observed, with tattooing leading to high antibody responses and subcutaneous DNA leading to high CD8 responses. We conclude that of the reporter proteins used, expression from DNA plasmids can best be assessed using tdTomato or luciferase. But, the disconnect between visible expression level and immunogenicity suggests that *in vivo* whole animal imaging of fluorescent proteins has limited utility for predicting DNA vaccine efficacy.

## Introduction

The use of DNA encoding antigen as a vaccine has many attractive features [1]. These include ease and speed of antigen development, temperature stability of the vaccine and similar patterns of post-translational modification to intracellular pathogens [2]. The ease of development of DNA vaccines is especially of interest for viral pathogens, for example influenza, that require regular updating to match circulating strains or rapid development to prevent fast spreading pandemics. However, whilst extremely effective in small animal models, DNA vaccines have failed to realise their potential in human clinical trials [3]. One factor limiting the progression of DNA vaccines into the clinic may be poor expression levels in human tissue after immunisation [4,5].

One approach to understand and improve DNA vaccine delivery is to track expression *in vivo* using fluorescent proteins. Since the discovery and development of green fluorescent protein [6], there has been a rainbow explosion of available proteins [7]. Fluorescent proteins have a number of characteristics that affect their efficacy as *in vivo* markers of expression, including wavelength, brightness and structure. The excitation and emission wavelengths of the protein used affects background fluorescence, blue or green fluorescent proteins tend to have higher levels of background because skin reflects light in the shorter/blue wavelengths of the spectrum, as an adaptation to protect against UV induced DNA damage [8]. To reduce background, fluorescent proteins have been designed in the red and far-red spectra [9–11], which promise better signal to noise ratios. A wider gap between excitation and emission can also reduce background by reducing the acquisition of reflected light from the excitation beam. The brightness (degree of fluorescence of the protein) is a function of a number of factors including the quantum yield—the ratio of the number of photons emitted to the number of photons absorbed (the maximum is 1.0); expression maturation and the extinction coefficient—a measure of how strongly the protein absorbs light at a given wavelength. The structure of the protein, particularly whether it is a monomer, dimer or tandem dimer can affect its behaviour *in vivo* and also has an effect on the cloning of the proteins as tandem repeats may make conventional PCR approaches challenging.

However, most of the studies used to characterise fluorescent proteins *in vivo* have either imaged injected cells overexpressing the protein [12] or directly injected protein [10]. In order to be a useful tool for the monitoring of gene expression after DNA delivery, the protein needs to have sufficient signal strength following de novo expression in tissue. The aim of the current study was to identify an effective red fluorescent protein for tracking DNA transgene expression *in vivo* and to explore how expression affects immunogenicity for vaccines. We assessed four fluorescent proteins (tdTomato, mCherry, Katushka and tdKatushka2) which have a range of excitation and emission characteristics, brightness and structures. We observed limited levels of expression *in vivo* with all the red proteins used, with tdTomato being the strongest. Different delivery routes led to different expression levels, with a stronger fluorescent signal after intramuscular delivery than subcutaneous or tattooing. The difference in visible expression levels did not appear to correlate with the outcome of infection following vaccination, with protection observed against influenza H1N1 infection when a DNA vaccine encoding haemagglutinin was delivered by either the intramuscular, subcutaneous or tattooing routes. This suggests that *in vivo* whole animal imaging of fluorescent proteins has limited utility for predicting DNA vaccine efficacy.

## Materials and Methods

### Plasmids expressing marker proteins

Plasmids expressing fluorescent proteins (Table 1) were obtained from the following commercial sources: tdTomato (Clontech: pCMV-tdTomato), mCherry (Clontech: pLVX-EF1α-IRES-mCherry), Katushka (Evrogen: pTurboFP635-C), tdKatushka2 (Addgene: 30181/ pTEC22) and firefly luciferase (Touchlight Genetics Ltd, Leatherhead, UK: proTLx Lux). Plasmids were maintained in *Escherichia coli* strain TOP10 and purified using Endo-Free giga kit (Qiagen, Manchester, UK).

**Table 1:** Fluorescent proteins used in the study.

Protein	Excitation maximum (nm)	Emission maximum (nm)	Quantum yield	Extinction coefficient	Brightness	Structure
**tdTomato** [9]	554	581	0.69	138000	95	Tandem dimer
**mCherry** [9]	587	610	0.22	72000	16	Monomer
**Katushka** [10]	588	635	0.34	65000	22.1	Dimer
**tdKatushka2** [11]	588	633	0.37	62500 x 2	25	Tandem Dimer

Values are taken from published studies (as indicated) and http://fccf.salk.edu/fluorochrometable.php.

### *In vitro* characterisation of fluorescent proteins

CHO-K1 (ATCC origin, Virginia, USA) cells were grown in DMEM (Gibco, Invitrogen, Paisley, UK) supplemented with 10% inactivated FCS, L-glutamate and penicillin and streptomycin. 5x10^5^ cells were seeded per well in a 24 well plate. 1μg of each plasmid was incubated for 30 minutes at room temperature with Lipofectamine 2000 Reagent (Invitrogen) before being added to the wells. *In vitro* imaging for expression of fluorescence was carried out using a Nikon Eclipse TE2000-S inverted microscope under bright light and a TxRed filter set (excitation 540–580nm and emission 600–660nm) at 24, 48 and 72 hours post transfection. The cells were also imaged with the *In vivo* Imaging System FX Pro (Bruker Biospin, Coventry, UK) and image analysis performed using Molecular Imaging SE software version 5.02 (Bruker), using the region of interest function and subtracting the mean of the control transfected wells.

### *In vivo* transfection

All experiments were carried out in accordance with Animals (Scientific Procedures) Act 1986 and the overall study approved by the Imperial College London Local Animal Welfare and Ethical Review Board (AWERB). 6–8 week old female BALB/c mice were purchased from Harlan (Quarriston, UK). For intramuscular (i.m.) delivery mice were injected with 50μg DNA in the anterior tibialis muscle of one leg, for subcutaneous (s.c.) delivery 50μg was injected in the epigastric region. Where used, the site was electroporated with a CUY21 EDIT system (BEX, Tokyo, Japan) with 5 successive 50-msec pulses of 150mV with 100-msec intervals, intramuscular electroporation was delivered by a needle type electrode, and subcutaneous electroporation was delivered by paddle electrode. For tattooing, fur was shaved from the leg of the mouse and each mouse had 10μl of 50μg DNA administered onto the skin and tattooing (Stealth Rotary Tattoo System, The Tattoo shop, Bolton, UK) was carried out at an oscillation frequency of 100Hz and needle depth of 0.5mm as described in [13], tattoo DNA was delivered without electroporation.

### *In vivo* imaging

For fluorescent images, on days 3, 5 and 7 after transfection the mice were imaged at excitation 550nm, emission 600nm or excitation 600nm, emission 700nm using the *In vivo* Imaging System FX Pro (Bruker Biospin, Coventry, UK). Prior to imaging mice were anaesthetised with inhaled isoflurane. Mice were imaged for 60 seconds. To avoid autofluorescence at the imaging site, mice were shaved and depilated using a commercially available depilation cream (Veet sensitive, Reckitt Benckiser, Hull, UK).

For luminescence, after transfection with plasmid encoding luciferase, expression was visualized following intraperitoneal injection of Rediject D-Luciferin (Perkin Elmer, Waltham, MA, USA) in accordance with manufacturer’s specification. Light emission was measured for 4 minutes without binning and an X-ray was taken for 30 seconds, the two images were overlaid using MI SE software. Relative luminescence was quantified by using the software’s region of interest (ROI) analysis function, with background levels set using control, untransfected animals.

### Immune response to influenza infection

Mice were immunized by the intramuscular, subcutaneous and tattooing routes with 5μg DNA expressing influenza haemagglutinin (HA) from A/England/195/2009. Mice were immunized in a prime boost regime with immunizations on day 0 and 14 and infection challenge on day 28. Protection against influenza infection following DNA vaccination was assessed as described previously [14]. H1N1 Influenza strain (A/England/195/2009) was grown in Madin-Darby Canine Kidney (MDCK) cells, in serum free DMEM supplemented with 1μg/ml trypsin [15]. Mice were infected intranasally with 5x10^5^ PFU influenza virus. Weight was measured daily to monitor disease severity. The harvesting of lung tissues was carried out as previously described [16]. Lungs were homogenized through 100μm cell strainers (BD, Oxford, UK) and washed through with a 1ml volume of RPMI. After the removal of the supernatants, cells were treated with ACK lysing buffer for 5 minutes and they were resuspended in RPMI. Cell viability was assessed by trypan blue exclusion, and total cell numbers were counted by disposable multiwell haemocytometer. For flow cytometry, prior to staining cells were blocked with CD16/32 (Fc Block, BD). For surface staining antibodies against the surface markers CD4, CD8, CD3, (BD) and the H-2K^d^ pentamer for the IYSTVASSL peptide (Proimmune, Oxford, UK) were added for 30 minutes on ice. Gating for lymphocytes was determined by back gating on CD3/CD8 double positive cells. Cells were analyzed on an LSR Fortessa flow cytometer (BD) collecting data on at least 50,000 events.

### Antigen specific ELISA

Serum antibodies specific to Influenza HA1 were measured using a standardized ELISA, modified from [17]. MaxiSorp 96-well plates (Nunc) were coated with 100μl of HA1 (A/England/195/2009, Life Technologies), or a combination of anti-murine lambda and kappa light chain specific antibodies (AbDSerotec, Oxford, UK) and incubated overnight at 4°C. Plates were washed and blocked with 1% bovine serum albumin (BSA) in PBS for 1 hour at 37°C. Sera were diluted in 1% BSA PBS and 100μl was added to each sample well. A titration of IgG standard was added to the kappa/lambda capture antibody coated wells. Plates were incubated for 1 hour at 37°C and washed before addition of goat anti-mouse IgG-HRP (Southern Biotech, Cambridge, UK) secondary antibody and incubated for 1 hour at 37°C. Plates were washed and developed with 50μl/well of KPL SureBlue TMB substrate (Insight Biotechnology, UK). The reaction was stopped after 5 min by adding 50μl/well 1 M H_2_SO_4_, and the absorbance read at 450 nm on a FLUOstar spectrophotometer (BMG LABTECH GmbH, Ortenberg, Germany). Absorbance values for the standard titration were fitted with a four parameter logistic curve and unknown values were interpolated using BMG Omega software.

### Statistical analysis

The appropriate statistical test was performed using GraphPad prism 5.01 (GraphPad Software Inc., La Jolla, CA, USA). Based on the type of data either a 2 way ANOVA with Bonferroni’s post test or a student’s T-test was performed.

## Results

### tdTomato yields the brightest response after *in vitro* transfection

**Fig 1:**
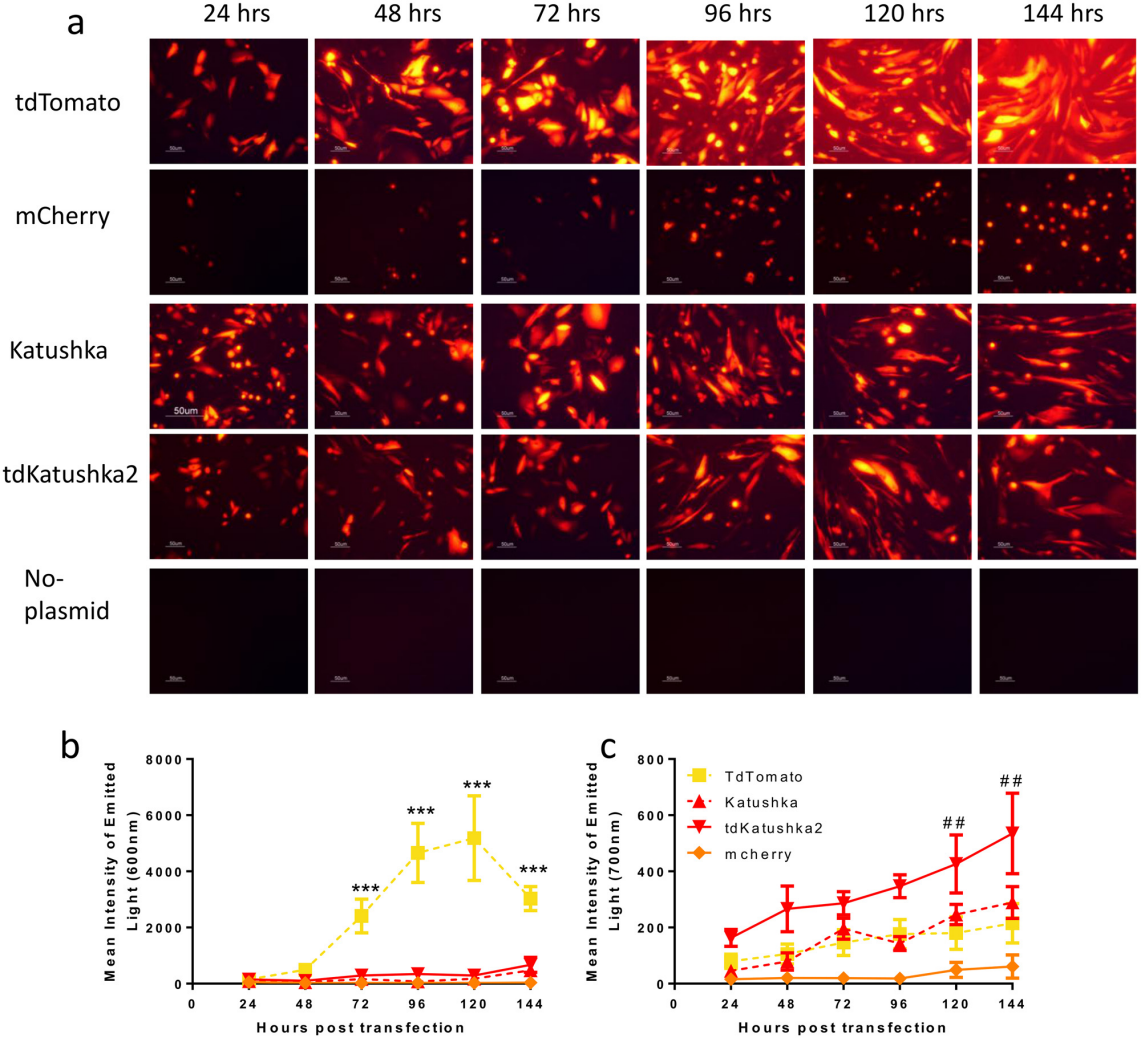
Comparison of red fluorescent proteins *in vitro*. CHO-k1 cells were transfected with 1μg DNA complexed with Lipofectamine. At various time points, cells were imaged by fluorescent microscopy—excitation 540–580nm and emission 600–660nm (a). Fluorescence intensity was measured by image analysis software at 550–600 nm (b) or 600–700 nm (c). Images in (a) representative of n = 3 experiments, points in b and c represent mean +/- SEM of 4 repeats, *** p<0.001 comparing tdTomato and other proteins in panel b, ## p<0.01 comparing tdKatushka2 and other proteins in panel c, by 2 way ANOVA.

We selected four fluorescent proteins with a range of excitation and emission characteristics, brightness and structures in the yellow-red range for assessment in this study (Table 1). The proteins selected were tdTomato, mCherry, Katushka and tdKatushka2. To initially screen for visible expression, we compared fluorescence after transfection of CHO-k1 cells *in vitro* over a six day time course. Cells were imaged using a Nikon Eclipse TE2000-S inverted microscope under bright light and a TxRed filter set (excitation 540–580nm and emission 600–660nm) (Fig 1a). All the proteins tested induced expression of red fluorescence detectable by microscopy, with a suggestion that tdTomato was brighter, however the excitation wavelength range used for microscopy was probably favourable to tdTomato. To more directly compare expression at appropriate wavelengths macroscopic images of the plates were taken using a Bruker *In-Vivo* MS FX Pro system at excitation 550nm and emission 600nm and quantified using imaging software (Fig 1b). Wells transfected with tdTomato were significantly brighter than those transfected with other proteins (p<0.001). When the plates were imaged at a higher fluorescence excitation (600nm) and emission (700nm) wavelength (Fig 1c), increased signal was observed for the red-shifted protein tdKatushka2, with significantly greater response than tdTomato at 120 and 144 hours after transfection (p<0.01). However the total intensity was lower than that observed for tdTomato at the lower wavelength. It was of note that there was a greater light signal for the tandem dimer tdKatushka2 than the dimer Katushka which may be because it is expressed more efficiently. Interestingly, the kinetic of the emitted light was different between the two wavelengths for unknown reasons.

### tdTomato yields the brightest response after *in vivo* delivery

**Fig 2:**
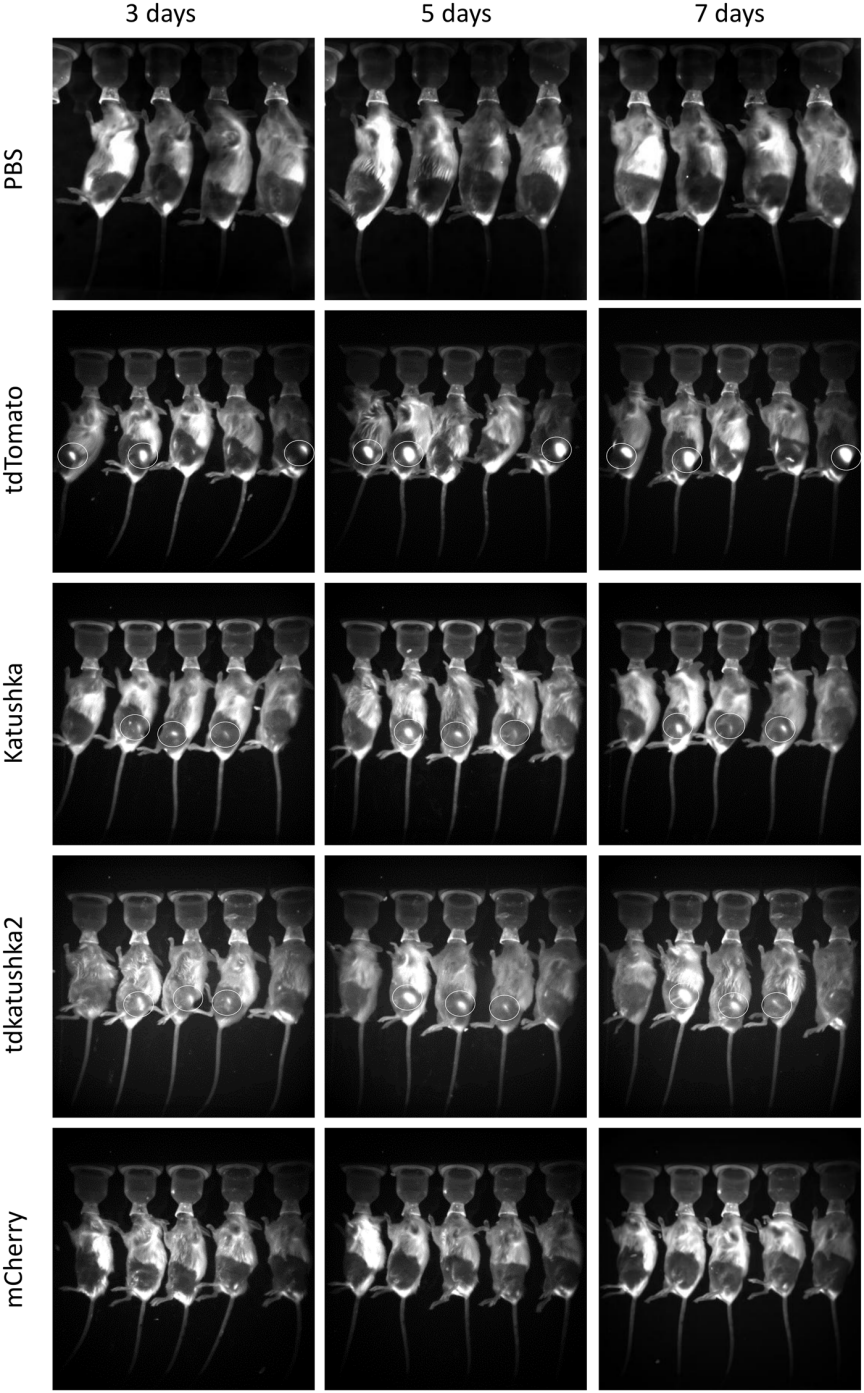
Comparison of red fluorescent proteins after intramuscular DNA delivery. Mice were injected i.m. with 50μg DNA in the anterior tibialis muscle and the site electroporated. At days 3, 5 and 7 after transfection, mice were imaged *in vivo* using an Imaging System at 550nm excitation and 600nm emission. Representative of n = 3 experiments. White circles indicate fluorescent protein expression.

**Fig 3:**
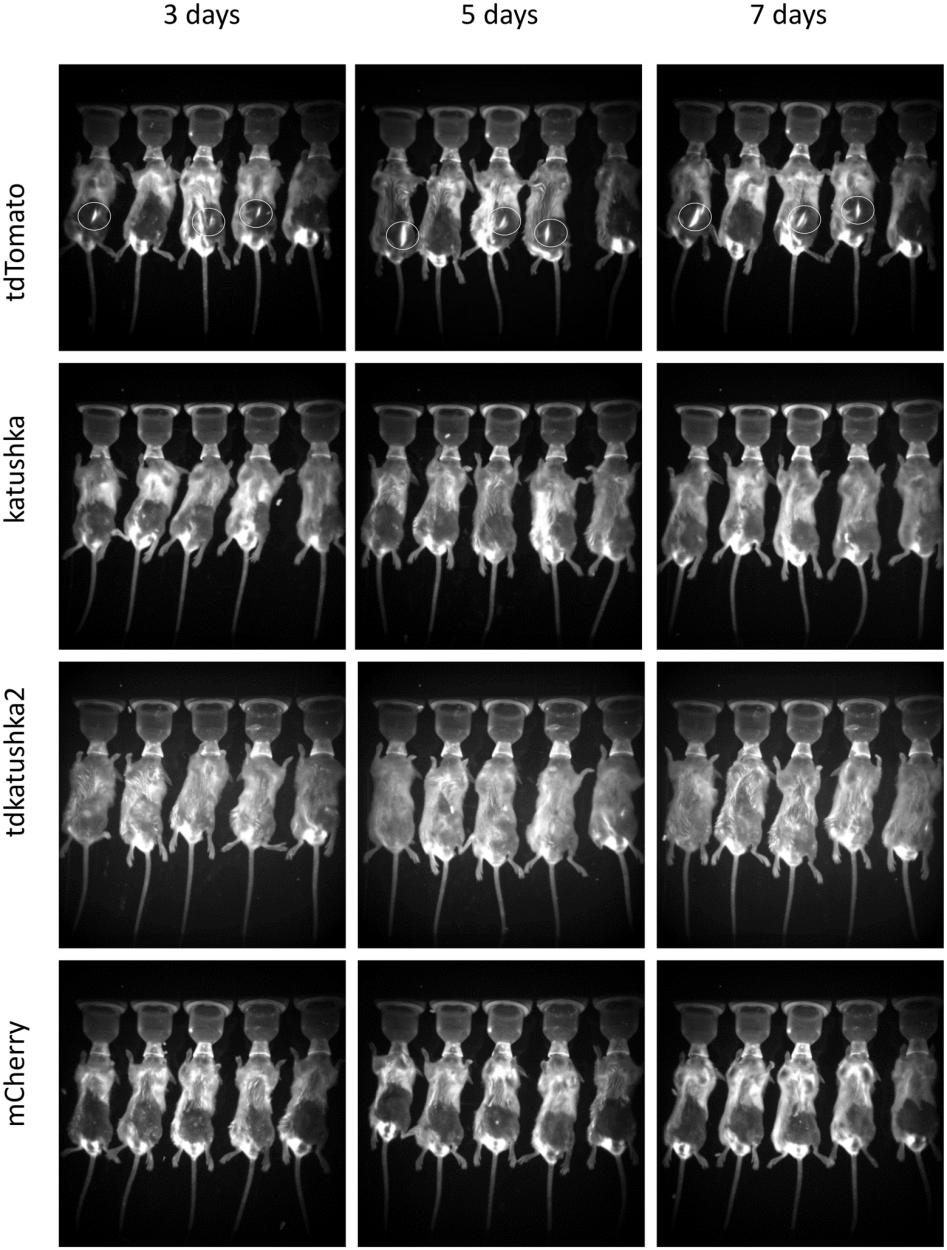
Comparison of red fluorescent proteins after subcutaneous DNA delivery. Mice were injected s.c. with 50μg DNA in the epigastric region and the site electroporated. At days 3, 5 and 7 after transfection, mice were imaged *in vivo* using an Imaging System at 550nm excitation and 600nm emission. Representative of n = 3 experiments. White circles indicate fluorescent protein expression.

The proteins were tested *in vivo* using three routes of delivery, intramuscular, subcutaneous and intradermal by tattooing of the skin. Delivery of DNA by tattooing of the skin was chosen as it has been shown to induce strong T and B cell responses [18,19]. The same dose of 50μg DNA was delivered by each route. Electroporation dramatically increases the expression of protein after *in vivo* DNA delivery [20] and was used for the intramuscular and subcutaneous routes. Animals were imaged at excitation 550nm and emission 600nm on days 3, 5 and 7 after gene delivery. Signal was detected following intramuscular delivery of DNA in the tdTomato (3 out of 5 animals), tdKatushka2 (3 out of 5 animals) and Katushka (3 out of 5 animals) groups, but not the mCherry group, of these tdTomato gave the strongest signal (Fig 2). Expression was only detectable after subcutaneous delivery of tdTomato (3 out of 5 animals, Fig 3) and not detectable after tattoo delivery (data not depicted). From this we conclude that tdTomato is the most effective protein for fluorescent imaging when delivered as a gene, but of limited use, with a number of limitations including heterogeneity of expression between animals in the same group and high background autofluorescence due to various sources of noise.

**Fig 4:**
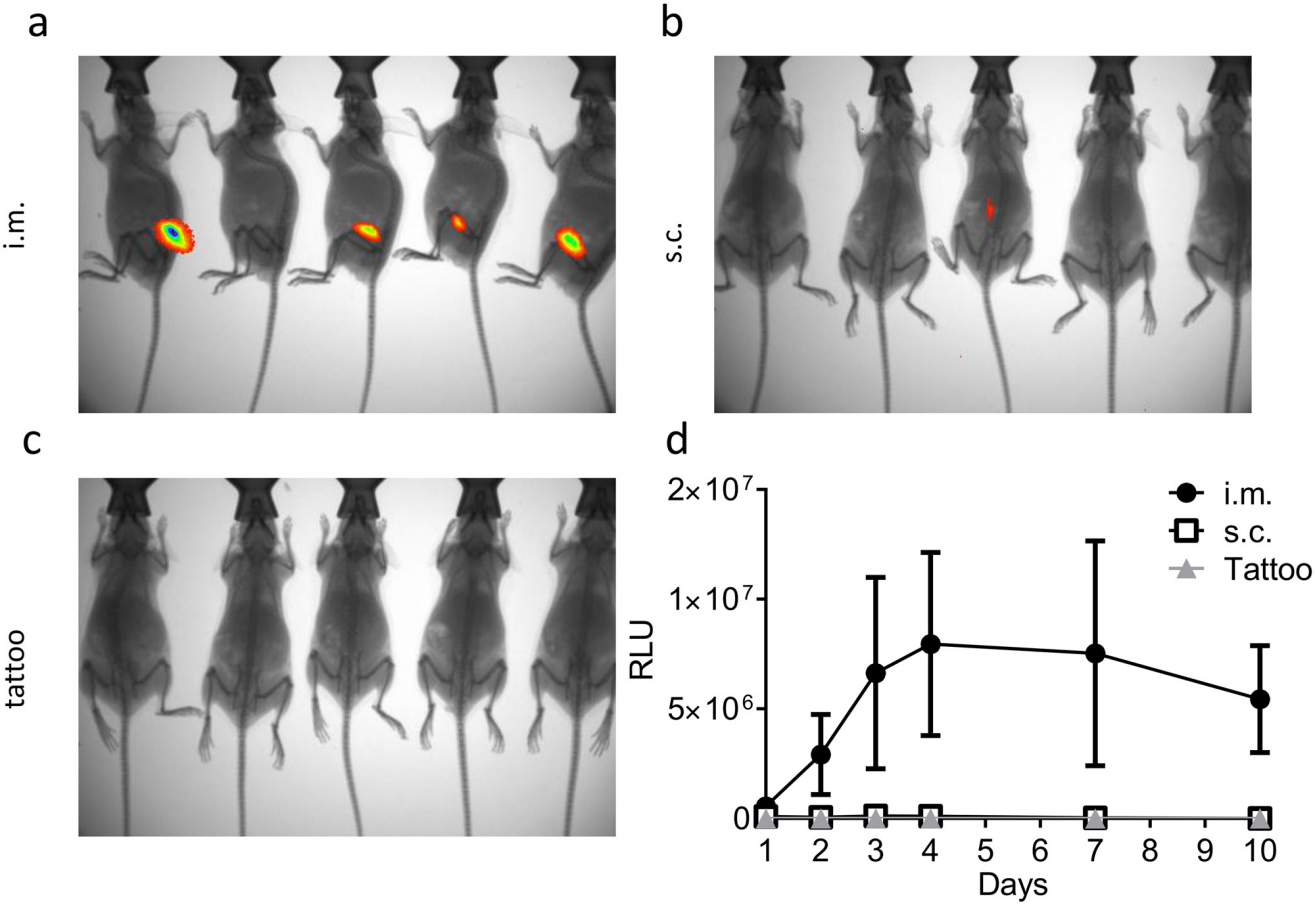
Comparison of luciferase expression after DNA by various routes. 50μg DNA encoding luciferase was delivered by the i.m., s.c. or tattooing routes. At various time points after transfection, mice were injected i.p. with Luciferin substrate and imaged *in vivo* using an Imaging System. Representative images from day 7 after transfection by the i.m. (a), s.c. (b) and tattoo (c) routes. Relative expression levels were quantified using image analysis software (d), points represent mean +/- SEM of n = 5 animals.

Since the signal was weak, even in the tdTomato group, we looked at luminescence as an alternative method of imaging. This has the advantage of being an enzymatic reaction and therefore can be amplified, it also has less background because it measures the direct release of photons rather than reflection of photons at a different wavelength. Luminescence quantification following delivery of the firefly luciferase gene has been used in a number of DNA vaccine studies [14,21]. Luciferase expression was detectable in 4 out of 5 mice after intramuscular delivery (Fig 4a) but only 1 out of 5 mice after subcutaneous delivery (Fig 4b), no luciferase signal was detected after tattoo DNA delivery (Fig 4c). Again, expression was quite variable, but the data suggests that the peak of expression was between 4 and 7 days after transfection (Fig 4d) as seen in previous studies [22].

### Immunisation by different routes alters the immune response to infection

**Fig 5:**
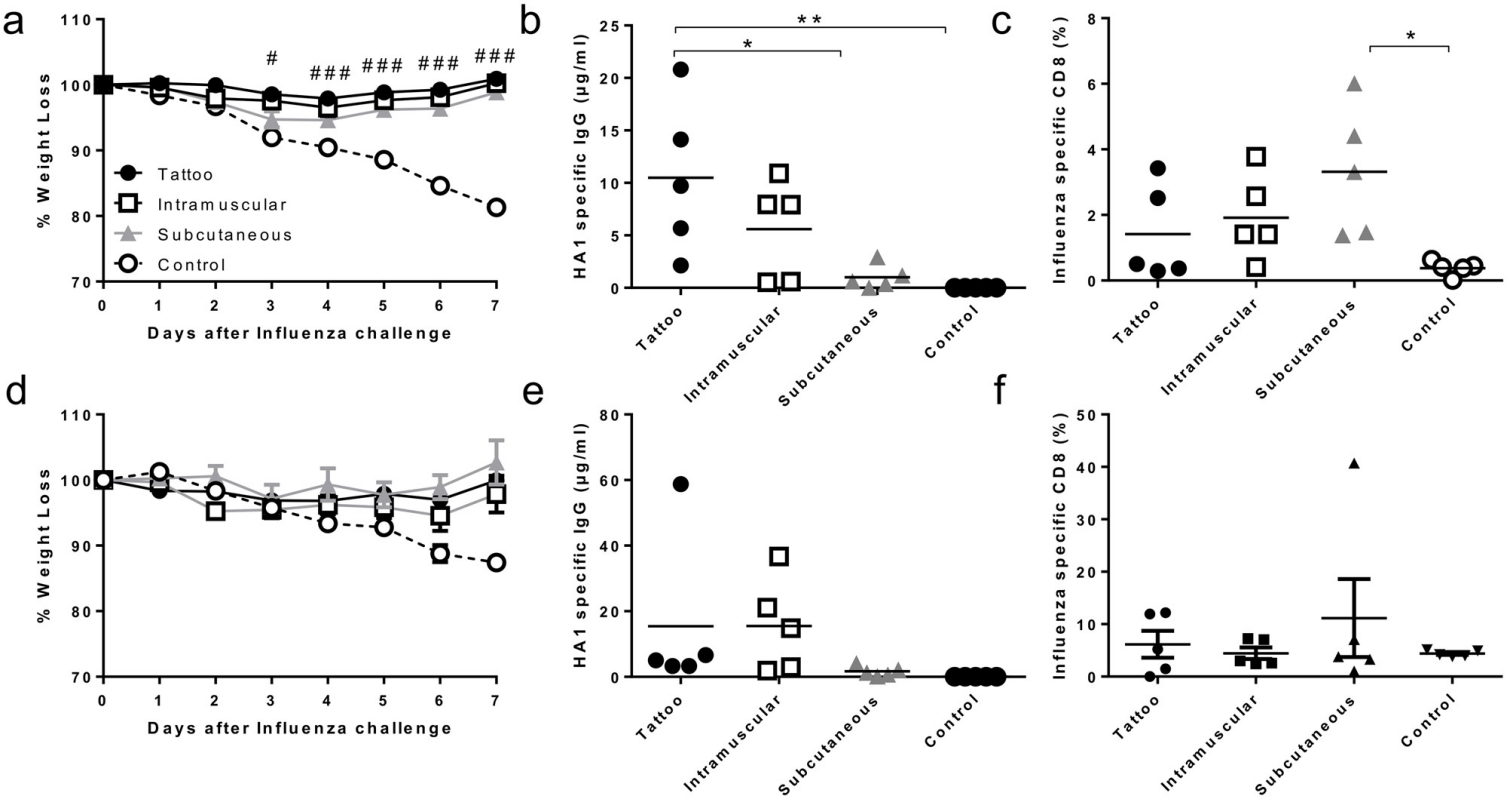
Comparison of immunogenicity after DNA by various routes. 5μg DNA encoding H1 haemagglutinin was delivered by the i.m., s.c. or tattooing routes on days 0 and 14. On day 28, mice were infected with 5x10^5^ H1N1 influenza in 100μL intranasally. Weight loss was monitored following infection (a), serum HA1 specific IgG (b), and lung influenza specific (pentamer stained) CD8+ T cells (c) were measured on day 7 after infection. In a subsequent study, mice were immunised with 5μg DNA encoding H1 haemagglutinin delivered by the i.m., s.c. or tattooing routes on days 0 and were infected on day 14 with 5x10^5^ H1N1 influenza in 100μl intranasally. Weight loss was monitored following infection (d), serum HA1 specific IgG (e), and lung influenza specific (pentamer stained) CD8+ T cells (f) were measured on day 7 after infection. Points represent n = 5 animals +/- SEM (a) or individuals (b, c) +/- SEM. # p<0.05, ### p<0.001 between control and other groups analysed by 2 way ANOVA (a), * p<0.05, ** p<0.001 as indicated, analysed by one way ANOVA and post test (b, c).

We wished to determine whether the level of gene expression estimated by imaging altered the outcome of DNA immunisation. From the imaging studies we inferred that expression was greatest after intramuscular injection followed by subcutaneous injection, with minimal expression after tattooing, the same routes were used in a vaccination study. Mice were immunised with 5μg plasmid DNA expressing haemagglutinin from influenza H1N1 (A/England/195/2009) via each of the three routes in a prime boost regime (two doses of DNA in total), prior to intranasal infection with a matched viral strain. Responses were compared to naïve control mice. All three groups of immunised mice were protected from influenza infection compared to naive mice, which lost significantly more weight on days 6 and 7 post infection (Fig 5a). Interestingly there were qualitative differences in the immune response to different routes of infection. Mice immunised by the tattooing route lost marginally less weight on days 3–6 than the subcutaneously immunised mice. Tattooing and intramuscular immunisation led to a strong antibody response, and the response after tattooing was significantly greater than that observed following subcutaneous (Fig 5b). However, there was a greater CD8 T cell response following subcutaneous immunisation (Fig 5c). Since all groups were protected after two vaccinations, we investigated whether differences in protection were observed after a single immunisation. Mice received a single immunisation with 5μg plasmid DNA expressing haemagglutinin from influenza H1N1 (A/England/195/2009) via each of the three routes prior to intranasal infection with influenza. As with the prime boost regime, a single immunisation with DNA by either of the routes led to protection against influenza infection (Fig 5d). However the qualitative differences between the routes of delivery were less pronounced after a single immunisation (Fig 5e and 5f). Therefore, the measurable level of protein expression by imaging may not determine the overall outcome of immunisation against influenza, but may alter the type of response seen.

## Discussion

The aim of the study was to compare DNA expression by *in vivo* imaging as a means to assess expression levels of DNA vaccines. A key finding was that all fluorescent proteins gave limited, heterogeneous signal measurable by *in vivo* imaging. Of the red fluorescent proteins tested in this study, tdTomato was the best for *in vivo* expression studies. This is the first study directly comparing the *in vivo* performance of these proteins when expressed from DNA. A previous comparison of red fluorescent proteins using transfected cells inserted into dead animals via capillary tubes [23], demonstrated that proteins with emission spectra >600nm are more effective because the emitted light is not absorbed by the tissues. In comparison, the current study looked at fluorescent proteins expressed from DNA directly injected into the animal which is more relevant to studying DNA vaccines (and gene therapy) than using cells that had been transfected prior to injection. Proteins with red shifted (higher emission) wavelengths did not give a stronger signal suggesting that brightness is the key variable for this application. There are other red, far-red and infrared fluorescent proteins available which may yield a stronger signal *in vivo* which were not included in this study [24], but none are theoretically as bright as tdTomato and some of the infrared proteins require the injection of additional cofactors to fluoresce. Of note, one technical issue with the study is that different promoters were used, tdTomato and Katushka were on a CMV promoter, mCherry was on an Ef1a promoter and tdKatushka2 was on a Mycobacterium Strong Promoter, which may affect expression efficiency.

Even when red proteins are used, there are a number of sources of auto-fluorescent noise that can occur within the experiment, including faeces, skin, fur and pen used to label the animals. Careful preparation of the animals and the use of strongly expressing/ fluorescing proteins can reduce this problem, but not completely mitigate it. We investigated whether luciferase could be a favourable alternative to fluorescent proteins for *in vivo* expression studies and it gave a cleaner signal. However, there are some limitations with luciferase including the requirement for oxygen to catalyse the reaction, which precludes use in deep tissue and the need to inject a substrate into the animals, which can increase the cost of the study. In the current study we used luciferase from firefly, *Photinus pyralis*, but other brighter and smaller luciferase proteins are being developed which may be more effective in this context including those derived from the sea pansy, *Renilla reniformis* and the deep sea shrimp, *Oplophorus gracilirostris* (NanoLuc) [25]. As we have previously seen, [26], there was considerable heterogeneity between animals in the visible expression levels using either fluorescent or luminescent proteins, which is a further limitation of these approaches.

Of the routes tested, intramuscular DNA delivery gave the greatest signal with both tdTomato and luciferase, suggesting the highest expression level, or at least the best retention of functional protein. Expression of sufficient functional reporter protein to be measured on the *in vivo* imager is most likely to be determined by the cells that take up and express the transgene. DNA delivered to the muscle, most likely enters myocytes which are energy rich, low turnover cells leading to higher expression levels. DNA delivered via the tattoo route enters the dermal space (because the depth of the tattoo delivery was 0.5 mm) where it may be taken up by adipocytes and fibroblasts which are less metabolically active than myocytes or enter antigen presenting cells which migrate away from the site of transfection and degrade the expressed antigen for presentation on MHC. Subcutaneous delivered DNA is probably taken up by a mixture of myocytes and adipocytes. A further consideration is that the induction of a cellular immune response to expressed transgene, leads to the targeting and clearance of transfected cells, limiting expression after DNA vaccination [27,28]. Reporter proteins also induce a self-limiting immune response leading to their clearance [29,30], particularly when the same animals are re-used.

There was a disconnect between observed expression level and immunogenicity, whilst we observed greater tdTomato and luciferase signal by the intramuscular than the subcutaneous or tattooing routes, all three routes appeared to be equally protective against influenza infection. However there were qualitative differences in the immune response. These studies suggest that the expression level required to induce an immune response is very much less than that required to be visualised by *in vivo* imaging. It also suggests that that DNA vaccine efficacy is more finessed than expression alone. Expression potentially reflects a minimum threshold over which a DNA vaccine needs to cross (akin to activation energy). In DNA vaccine clinical trials it is conceivable that the requisite level of expression was not achieved. This is probably due to scale up issues, both with vaccine volume and vaccine amount [31]. Approaches are being explored to increase expression in human DNA vaccines, including electroporation [20], novel formulations [32] and novel DNA constructs [14]. But above a certain expression threshold, DNA vaccine delivered antigens will act like conventional protein antigens with other factors affecting the immune response, including antigen design, route of delivery, formulation and adjuvants. Therefore, attention needs to be paid to enhancing immunogenicity in addition to improving expression in the target tissue.

In this study we observed a qualitative change in immune response between the different routes of DNA delivered, with greater antibody responses when DNA was delivered by tattooing, greater T cell responses with subcutaneous delivery and a balanced response to intramuscular delivered DNA. Immunogenicity is affected by a number of factors including expression level and the types of antigen presenting cells that process the antigen, there are a number of studies that explore the impact of different populations of antigen presenting cells in different layers of the skin and the muscle [33]. A broad comparison of the effect of route of delivery of DNA vaccines in mouse and macaques showed differences between the routes [34]. However, a recent clinical trial comparing DNA vaccines delivered by the intramuscular, subcutaneous or intradermal routes found no difference in immunogenicity, but found mildly greater reactogenicity after intradermal or subcutaneous delivery [35]. It was of note that tattoo delivered DNA gave no visible signalling by imaging, but gave the strongest antibody response. The induction of antibody responses to DNA vaccines is often poor and it may be due to retention of the protein in the cell preventing the B cells from encountering the antigen. Tattooing may induce a small wave of protein expression [18] simultaneously damaging the cells leading to cell death and release of the protein into a space where it can interact with B cells. As antigen responses to DNA vaccines in clinical trials have thus far been poor, it would be of interest to see if tattooing can improve the response in humans. However, as with other DNA vaccine approaches, there may be issues of scale up into human studies, in murine studies 1 cm^2^ surface area represents a considerable proportion of the mouse leg and it is unclear whether a human vaccine would need to cover a scaled up area for equivalent effect, for example in rhesus macaques a 30 cm^2^ area was used [36].

In this study we explored the impact of the expression level and route of delivery on the immune response to DNA vaccines. We found that tdTomato and luciferase were the best reporter proteins and that intramuscular delivery led to the greatest expression of transgene. This may be of value both for DNA vaccine development and also other DNA delivery applications, for example gene therapy. However, the immunogenicity of the delivered vaccine was not solely determined by the expression level of the antigen and antigen expression as measured by *in vivo* imaging did not predict the intensity of the immune response. Therefore increasing the immunogenicity of the expressed antigen is therefore also critical in the development of an effective DNA vaccine in man.

## Supporting Information

S1 Table Excel format raw data file for all studies described in MS. (XLSX)

## References

[1] Kutzler MA, Weiner DB (2008) DNA vaccines: ready for prime time? Nat Rev Genet 9: 776–788. 10.1038/nrg2432 18781156 PMC4317294

[2] Shedlock DJ, Weiner DB (2000) DNA vaccination: antigen presentation and the induction of immunity. J Leukoc Biol 68: 793–806. 11129646

[3] Tregoning JS, Kinnear E (2014) Using Plasmids as DNA Vaccines for Infectious Diseases. Microbiology Spectrum 2. 10.1128/microbiolspec.PLAS-0028-2014 26104452

[4] Romero NB, Braun S, Benveniste O, Leturcq F, Hogrel JY, Morris GE, et al (2004) Phase I study of dystrophin plasmid-based gene therapy in Duchenne/Becker muscular dystrophy. Hum Gene Ther 15: 1065–1076. 15610607 10.1089/hum.2004.15.1065

[5] Comerota AJ, Throm RC, Miller KA, Henry T, Chronos N, Laird J, et al (2002) Naked plasmid DNA encoding fibroblast growth factor type 1 for the treatment of end-stage unreconstructible lower extremity ischemia: preliminary results of a phase I trial. J Vasc Surg 35: 930–936. 12021709 10.1067/mva.2002.123677

[6] Shimomura O, Johnson FH, Saiga Y (1962) Extraction, purification and properties of aequorin, a bioluminescent protein from the luminous hydromedusan, Aequorea. J Cell Comp Physiol 59: 223–239. 13911999 10.1002/jcp.1030590302

[7] Chudakov DM, Matz MV, Lukyanov S, Lukyanov KA (2010) Fluorescent proteins and their applications in imaging living cells and tissues. Physiol Rev 90: 1103–1163. 10.1152/physrev.00038.2009 20664080

[8] Weissleder R, Ntziachristos V (2003) Shedding light onto live molecular targets. Nat Med 9: 123–128. 12514725 10.1038/nm0103-123

[9] Shaner NC, Campbell RE, Steinbach PA, Giepmans BN, Palmer AE, Tsien RY (2004) Improved monomeric red, orange and yellow fluorescent proteins derived from Discosoma sp. red fluorescent protein. Nat Biotechnol 22: 1567–1572. 15558047 10.1038/nbt1037

[10] Shcherbo D, Merzlyak EM, Chepurnykh TV, Fradkov AF, Ermakova GV, Solovieva EA, et al (2007) Bright far-red fluorescent protein for whole-body imaging. Nat Methods 4: 741–746. 17721542 10.1038/nmeth1083

[11] Shcherbo D, Murphy CS, Ermakova GV, Solovieva EA, Chepurnykh TV, Shcheglov AS, et al (2009) Far-red fluorescent tags for protein imaging in living tissues. Biochem J 418: 567–574. 10.1042/BJ20081949 19143658 PMC2893397

[12] Deliolanis NC, Kasmieh R, Wurdinger T, Tannous BA, Shah K, Ntziachristos V (2008) Performance of the red-shifted fluorescent proteins in deep-tissue molecular imaging applications. J BiomedOpt 13: 044008 10.1117/1.2967184 19021336 PMC2749214

[13] Chiu YN, Sampson JM, Jiang X, Zolla-Pazner SB, Kong XP (2012) Skin tattooing as a novel approach for DNA vaccine delivery. J Vis Exp. 10.3791/50032 PMC3490318 23117298

[14] Walters AA, Kinnear E, Shattock RJ, McDonald JU, Caproni LJ, Porter N, et al (2014) Comparative analysis of enzymatically produced novel linear DNA constructs with plasmids for use as DNA vaccines. Gene Ther 21: 645–652. 10.1038/gt.2014.37 24830436 PMC4082409

[15] Elleman CJ, Barclay WS (2004) The M1 matrix protein controls the filamentous phenotype of influenza A virus. Virology 321: 144–153. 15033573 10.1016/j.virol.2003.12.009

[16] Tregoning JS, Yamaguchi Y, Wang B, Mihm D, Harker JA, Bushell ES, et al (2010) Genetic susceptibility to the delayed sequelae of neonatal respiratory syncytial virus infection is MHC dependent. J Immunol 185: 5384–5391. 10.4049/jimmunol.1001594 20921522

[17] Donnelly L, Curran RM, Tregoning JS, McKay PF, Cole T, Morrow RJ, et al (2011) Intravaginal immunization using the recombinant HIV-1 clade-C trimeric envelope glycoprotein CN54gp140 formulated within lyophilized solid dosage forms. Vaccine 29: 4512–4520. 10.1016/j.vaccine.2011.04.023 21514349 PMC3120965

[18] Bins AD, Jorritsma A, Wolkers MC, Hung CF, Wu TC, Schumacher TN, et al (2005) A rapid and potent DNA vaccination strategy defined by in vivo monitoring of antigen expression. Nat Med 11: 899–904. 15965482 10.1038/nm1264

[19] Pokorna D, Rubio I, Muller M (2008) DNA-vaccination via tattooing induces stronger humoral and cellular immune responses than intramuscular delivery supported by molecular adjuvants. Genet Vaccines Ther 6: 4 10.1186/1479-0556-6-4 18257910 PMC2267179

[20] Gothelf A, Gehl J (2012) What you always needed to know about electroporation based DNA vaccines. Hum Vaccin Immunother 8: 1694–1702. 10.4161/hv.22062 23111168 PMC3601144

[21] Geiben-Lynn R, Greenland JR, Frimpong-Boateng K, Letvin NL (2008) Kinetics of recombinant adenovirus type 5, vaccinia virus, modified vaccinia ankara virus, and DNA antigen expression in vivo and the induction of memory T-lymphocyte responses. Clin Vaccine Immunol 15: 691–696. 10.1128/CVI.00418-07 18272665 PMC2292663

[22] Gothelf A, Eriksen J, Hojman P, Gehl J (2010) Duration and level of transgene expression after gene electrotransfer to skin in mice. Gene Ther 17: 839–845. 10.1038/gt.2010.35 20376097

[23] Deliolanis NC, Kasmieh R, Wurdinger T, Tannous BA, Shah K, Ntziachristos V (2008) Performance of the red-shifted fluorescent proteins in deep-tissue molecular imaging applications. J Biomed Opt 13: 044008 10.1117/1.2967184 19021336 PMC2749214

[24] Shcherbakova DM, Verkhusha VV (2013) Near-infrared fluorescent proteins for multicolor in vivo imaging. Nat Methods 10: 751–754. 10.1038/nmeth.2521 23770755 PMC3737237

[25] Hall MP, Unch J, Binkowski BF, Valley MP, Butler BL, Wood MG, et al (2012) Engineered luciferase reporter from a deep sea shrimp utilizing a novel imidazopyrazinone substrate. ACS Chem Biol 7: 1848–1857. 10.1021/cb3002478 22894855 PMC3501149

[26] Walters AA, Kinnear E, Shattock RJ, McDonald JU, Caproni LJ, Porter N, et al (2014) Comparative analysis of enzymatically produced novel linear DNA constructs with plasmids for use as DNA vaccines. Gene Ther. 10.1038/gt.2014.37 PMC4082409 24830436

[27] Geiben-Lynn R, Greenland JR, Frimpong-Boateng K, van Rooijen N, Hovav AH, Letvin NL (2008) CD4+ T lymphocytes mediate in vivo clearance of plasmid DNA vaccine antigen expression and potentiate CD8+ T-cell immune responses. Blood 112: 4585–4590. 10.1182/blood-2008-06-165803 18784371 PMC2597129

[28] Greenland JR, Geiben R, Ghosh S, Pastor WA, Letvin NL (2007) Plasmid DNA vaccine-elicited cellular immune responses limit in vivo vaccine antigen expression through Fas-mediated apoptosis. J Immunol 178: 5652–5658. 17442948 10.4049/jimmunol.178.9.5652 PMC2262927

[29] Limberis MP, Bell CL, Wilson JM (2009) Identification of the murine firefly luciferase-specific CD8 T-cell epitopes. Gene Ther 16: 441–447. 10.1038/gt.2008.177 19129859 PMC10694863

[30] Han WG, Unger WW, Wauben MH (2008) Identification of the immunodominant CTL epitope of EGFP in C57BL/6 mice. Gene Ther 15: 700–701. 10.1038/sj.gt.3303104 18288211

[31] Leamy VL, Martin T, Mahajan R, Vilalta A, Rusalov D, Hartikka J, et al (2006) Comparison of rabbit and mouse models for persistence analysis of plasmid-based vaccines. Hum Vaccin 2: 113–118. 17012905 10.4161/hv.2836

[32] Greenland JR, Letvin NL (2007) Chemical adjuvants for plasmid DNA vaccines. Vaccine 25: 3731–3741. 17350735 10.1016/j.vaccine.2007.01.120

[33] Romani N, Flacher V, Tripp CH, Sparber F, Ebner S, Stoitzner P (2012) Targeting skin dendritic cells to improve intradermal vaccination. Curr Top Microbiol Immunol 351: 113–138. 10.1007/82_2010_118 21253784 PMC4285659

[34] McCluskie MJ, Brazolot Millan CL, Gramzinski RA, Robinson HL, Santoro JC, Fuller JT, et al (1999) Route and method of delivery of DNA vaccine influence immune responses in mice and non-human primates. Mol Med 5: 287–300. 10390545 PMC2230426

[35] Enama ME, Ledgerwood JE, Novik L, Nason MC, Gordon IJ, Holman L, et al (2014) Phase I randomized clinical trial of VRC DNA and rAd5 HIV-1 vaccine delivery by intramuscular (i.m.), subcutaneous (s.c.) and intradermal (i.d.) administration (VRC 011). PLoS One 9: e91366 10.1371/journal.pone.0091366 24621858 PMC3951381

[36] Verstrepen BE, Bins AD, Rollier CS, Mooij P, Koopman G, Sheppard NC, et al (2008) Improved HIV-1 specific T-cell responses by short-interval DNA tattooing as compared to intramuscular immunization in non-human primates. Vaccine 26: 3346–3351. 10.1016/j.vaccine.2008.03.091 18467010

[37] Ramakrishnan L, Federspiel NA, Falkow S (2000) Granuloma-specific expression of Mycobacterium virulence proteins from the glycine-rich PE-PGRS family. Science 288: 1436–1439. 10827956 10.1126/science.288.5470.1436

